# Supplementary material for: Proximal femoral nail antirotation versus InterTan nail for the treatment of intertrochanteric fractures: A systematic review and meta-analysis
Source: PLoS One. 2024 Jul 9;19(7):e0304654. doi: 10.1371/journal.pone.0304654 (PMC11233004; doi:10.1371/journal.pone.0304654)
Supplement: S1 Table — (DOCX) [file pone.0304654.s002.docx]

**Table 1** Characteristics of included literature studies

| Author | Study Design | Country | Year | Group | Patients | Age（years） | Gender（M/F） | Outcomes | Newcastle-Ottawa-Scale |
| --- | --- | --- | --- | --- | --- | --- | --- | --- | --- |
| Duramaz^8^ | Retrospective | Turkey | 2019 | PFNA | 100 | 61.01 ± 16.6 | 132/171 | ⑴⑵⑶⑸⑹⑺⑼ | 7 |
|  |  |  |  | Inter- TAN | 86 | 61.5±15.8 |  |  |  |
| Gavaskar^9^ | Retrospective | India | 2018 | PFNA | 50 | 78±8 | 21/29 | ⑸⑹⑺⑼ | 8 |
|  |  |  |  | Inter- TAN | 50 | 77±7 | 21/29 |  |  |
| Guo^10^ | Retrospective | China | 2021 | PFNA | 38 | NA | NA | ⑴⑶⑷ | 5 |
|  |  |  |  | Inter- TAN | 40 |  |  |  |  |
| Imerci^11^ | Retrospective | Turkey | 2018 | PFNA | 33 | 54.64±18.94 | 19/14 | ⑸⑹⑻⑼ | 7 |
|  |  |  |  | Inter- TAN | 36 | 57.86±22 | 23/13 |  |  |
| Makki^12^ | Retrospective | UK | 2015 | PFNA | 36 | 80±11.75 | 7/29 | ⑻ | 6 |
|  |  |  |  | Inter- TAN | 22 | 78±10.44 | 9/13 |  |  |
| Seyhan^13^ | Prospective | Turkey | 2015 | PFNA | 43 | 75.91±13.77 | 11/32 | ⑴⑵⑸⑺⑻⑼ | 8 |
|  |  |  |  | Inter- TAN | 32 | 75.34±13.52 | 8/24 |  |  |
| Wang^14^ | Retrospective | China | 2013 | PFNA | 36 | 76.8±9.5 | 17/19 | ⑴⑶⑸⑻⑼ | 6 |
|  |  |  |  | Inter- TAN | 20 | 73.5±11.3 | 11/9 |  |  |
| Yang^15^ | Retrospective | China | 2017 | PFNA | 51 | 79.3±7.4 | 6/45 | ⑴⑶ | 6 |
|  |  |  |  | Inter- TAN | 17 | 76.2±5.8 | 7/10 |  |  |
| Yu^16^ | Retrospective | China | 2016 | PFNA | 72 | 74.2±9.1 | 32/40 | ⑴⑵⑶⑷⑸⑺⑻⑼ | 7 |
|  |  |  |  | Inter- TAN | 75 | 75.2±8.8 | 35/40 |  |  |
| Zehir^17^ | Retrospective | Turkey | 2015 | PFNA | 96 | 77.22±6.82 | 37/59 | ⑴⑵⑶⑸⑺⑻ | 7 |
|  |  |  |  | Inter- TAN | 102 | 76.86±6.74 | 39/63 |  |  |
| Zhang^18^ | Prospective | Turkey | 2013 | PFNA | 56 | 72.4±8.7 | 19/37 | ⑴⑵⑶⑷⑸⑹⑺⑼ | 8 |
|  |  |  |  | Inter- TAN | 57 | 72.9±7.6 | 23/34 |  |  |
| Zhang^19^ | Retrospective | China | 2017 | PFNA | 139 | NA | 53/86 | ⑴⑶⑸⑹⑺⑼ | 7 |
|  |  |  |  | Inter- TAN | 144 |  | 64/80 |  |  |
| Zhang(a)^20^ | Retrospective | China | 2017 | PFNA | 64 | 73.3±6.5 | 25/39 | ⑴⑵⑶⑸⑻⑼ | 7 |
|  |  |  |  | Inter- TAN | 49 | 74.2±5.4 | 20/29 |  |  |
| Zhang(b)^21^ | Retrospective | China | 2017 | PFNA | 88 | 74.6±6.3 | 34/54 | ⑸⑺ | 7 |
|  |  |  |  | Inter- TAN | 86 | 72.7±7.6 | 30/56 |  |  |
| Zhang^22^ | Retrospective | China | 2018 | PFNA | 164 | NA | 77/87 | ⑴⑶⑸⑺⑼ | 8 |
|  |  |  |  | Inter- TAN | 162 |  | 73/89 |  |  |

Abbreviations:(1)Duration of surgery, (2)Fluoroscopy time, (3)Blood loss, (4)Hospital stay, (5)Harris hip score(HHS), (6)Good reduction quality, (7)Tip-apex distance(TAD),(8)Union of bone,(9)Complications
