# Supplementary material for: Proximal femoral nail antirotation versus InterTan nail for the treatment of intertrochanteric fractures: A systematic review and meta-analysis
Source: PLoS One. 2024 Jul 9;19(7):e0304654. doi: 10.1371/journal.pone.0304654 (PMC11233004; doi:10.1371/journal.pone.0304654)
Supplement: Attachment — Submitted filename: Response to Reviewers.docx [file pone.0304654.s003.docx]

Dear Reviewers,

Thank you for your valuable feedback on our manuscript titled "Proximal Femoral Nail Antirotation versus InterTan Nail for the treatment of intertrochanteric fractures: a systematic review and meta-analysis." We appreciate the time and effort you have taken to review our work, and we are grateful for your insightful comments. We have carefully considered your suggestions, and we would like to address them in our revised manuscript.

Reviewer #1:

1. Clarity of Findings: We agree that further clarification of certain aspects of the findings would enhance the manuscript. In the revised version, we will expand on the implications of postoperative complications on clinical outcomes, providing a more detailed discussion of how these complications may affect patient recovery and long-term outcomes.

2. Language and Grammar: We apologize for any inconsistencies in language and grammar. We have carefully reviewed the entire manuscript and made necessary corrections to improve readability and clarity.

3. Data Availability Statement: We understand the importance of data availability and have ensured that all data underlying the findings of this meta-analysis, including raw data points, are fully available as per PLOS ONE's data policy. The data used in our study are sourced from published journal articles, and all cited articles have been thoroughly listed in the text as well as the reference list. Since these data are openly available secondary resources, no additional data access permissions or application processes are required. However, for further information or verification of the data, interested readers are encouraged to refer to the corresponding original literature. If there are any questions or need for further assistance, please feel free to contact us via email.

Reviewer #2:

1. Abstract and Study Objectives: We have revised the abstract to provide a brief introduction and merged it with the study objectives, as suggested.

2. Reporting System and Registration: We used the Preferred Reporting Items for Systematic Reviews and Meta-Analyses (PRISMA) statement to guide the reporting of our review, and we have registered it on PROSPERO. We have added this information to the revised manuscript.

3. Study Selection and Data Extraction: We apologize for the missing information on study selection and data extraction. In the revised manuscript, we have added detailed descriptions of these processes, including the search strategy, inclusion and exclusion criteria, and the methods used for data extraction.

Reviewer #3:

1. Exclusion of Randomized Controlled Trials (RCTs): We acknowledge the crucial role of randomized controlled trials (RCTs) in evaluating the efficacy of treatments. However, in our initial review, we did not identify any RCTs that satisfied our stringent inclusion criteria, possibly due to the limited availability of RCTs specifically addressing the treatment of intertrochanteric fractures with PFNA and InterTan, or because the designs of these studies did not align with our specific requirements.Despite this, we emphasize the significant value of RCTs in assessing treatment effects and have highlighted this in the discussion section of our manuscript. We remain vigilant in monitoring the latest research in this field and will strive to include RCTs in our future studies when available.

2. Subgroup Analysis: Regarding the issue of subgroup analysis, we are well aware of its significance in enhancing the relevance and applicability of research results to different patient populations. However, unfortunately, the detailed data on patient demographics or fracture characteristics in the existing literature are incomplete, which prevents us from conducting a systematic subgroup analysis. We have attempted to contact the authors of the original literature to obtain more data, but have been unsuccessful. Nevertheless, we have emphasized this limitation in our paper and will pay more attention to the collection and analysis of relevant data in future studies.

Once again, we appreciate the time and effort spent by the reviewers in evaluating our manuscript. We believe that the revisions addressed in this response have significantly improved our work, and we hope that it is now suitable for publication.

Sincerely,

Changsheng-Liao
